# Evaluating the suitability of granular anammox biomass for nitrogen removal from vegetable tannery wastewater

**DOI:** 10.1007/s10532-023-10017-6

**Published:** 2023-02-17

**Authors:** C. Polizzi, T. Lotti, A. Ricoveri, G. Mori, D. Gabriel, G. Munz

**Affiliations:** 1 Department of Civil and Environmental Engineering, University of Florence, Via di S. Marta, 3, 50139 Florence, Italy; 2 Consorzio Cuoiodepur S.p.A, Via Arginale Ovest, 81, 56020 San Romano, Pisa Italy; 3 GENOCOV Research Group, Department of Chemical, Biological and Environmental Engineering, Escola d’Enginyeria, Universitat Autònoma de Barcelona, 08193 Bellaterra, Spain

**Keywords:** Anammox granular biomass, Vegetable tannery wastewater, Salinity, Tannins, Inhibition, Nitrogen removal

## Abstract

In the present study, the potential inhibitory effect of biologically pre-treated vegetable tannery wastewater (TW) on anammox granular biomass was evaluated. Beside high organic and chemicals load, vegetable TW are characterised by high salinity and high tannins concentration, the latter belonging to a group of bio-refractory organic compounds, potentially inhibitory for several bacterial species. Recalcitrant tannin-related organic matters and salinity were selected as the two potential inhibitory factors and studied either for their separate and combined effect. Parallel batch tests were performed, with biomass acclimated and non-acclimated to salinity, testing three different conditions: non-saline control test with non-acclimated biomass (CT); saline control test with acclimated biomass (SCT); vegetable tannery wastewater test with acclimated biomass (TWT). Compared with non-saline CT, the specific anammox activity in tests SCT and TWT showed a reduction of 28 and 14%, respectively, suggesting that salinity, at conductivity values of 10 mS/cm (at 25 °C), was the main impacting parameter. As a general conclusion, the study reveals that there is no technical limitation for the application of the anammox process to vegetable TW, but preliminary biomass acclimation as well as regular biomass activity monitoring is recommended in case of long-term applications. To the best of our knowledge, this is the first work assessing the impact of vegetable TW on anammox biomass.

**Supplementary Information:**

The online version contains supplementary material available at 10.1007/s10532-023-10017-6.

## Introduction

Tannery industry produces significant amounts of highly polluted wastewaters and industrial facilities are present all over the world, in developed and developing countries. Leather processing comprises multiple steps during which raw leather undergoes chemical-physical treatments aimed at stabilizing the organic matter and conferring the required physical properties to the final product. Two types of tanning process are usually adopted: Chromium(III)-based and vegetable-tannin-based, the former covering the vast majority of leather processing worldwide. The derived wastewaters are characterized by high concentrations of nitrogen (as ammonium and organic nitrogen), organic compounds, sulphur (as sulphide and sulphate), salts and chemicals (Mannucci et al. 2010; Saxena et al. 2016). The high content in chlorides is due to the fact that salted flesh remains the most cost-effective manner to preserve the raw material prior to its processing.

Depending on national regulations on effluent quality, nutrient removal can be required and biological treatments are typically adopted in order to achieve stringent discharge limits on nitrogen and carbonaceous compounds. Primary and tertiary treatments are also crucial for the removal of suspended and recalcitrant/colloidal matter, respectively (Saxena et al. 2016). The high salinity of TW is an important aspect that needs to be considered when biological treatments are adopted. Its adverse effect on biomass activity is further exacerbated by the fact that wastewater characteristics may vary significantly over the year according to the industrial activity (e.g. vacation periods and market fluctuations), exposing activated sludge biomass to salinity and load gradients. When vegetable or synthetic tannins are used in the production process, further drawbacks may arise in the biological treatment unit, due to their bio-recalcitrant and potentially inhibitory nature. Tannins inhibition on bacterial activities has been reported by Munz et al. (2009) and Nelson et al. (1997), among others. In conventional activated sludge systems treating vegetable TW, nitrifying bacteria have been reported as the most sensitive microbial population to high salinity and tannins gradients and low maximum growth rate are reported as an aggregate result of the inhibitory effect of compounds related to the tanning process (Moussa et al. 2006; Munz et al. 2009; Szpyrkowicz and Kaul 2004).

The wastewater treatment plant managed by Consorzio Cuoiodepur S.p.A. (San Romano, PI) is a large facility treating municipal wastewater and industrial wastewater generated by the vegetable-tannery district located in the Italian region of Tuscany, representing one of the most important district for vegetable-tanned leather production in Europe. Conventional primary, secondary and tertiary treatment are currently adopted to comply with Italian regulation for discharge. A schematic of the current plant configuration is presented in figure S.1 (supplementary material). In the perspective of WWTP rethinking towards energy self-sustainability and resource valorisation, anaerobic and autotrophic processes are gaining attention as alternatives to TW conventional activated sludge systems. As an example, innovative treatment lines might integrate Carbon (C), Nitrogen (N) and Sulphur (S) cycles in a newly conceived synergy exploiting anaerobic treatments, anammox process and autotrophic denitrification (Kalyuzhnyi et al. 2006). Specifically, COD load could be valorised through anaerobic digestion and the N-rich downstream digestate could be treated through the anammox process (AMX); nitrite required for AMX could be supplied by partial autotrophic denitrification achieving simultaneous sulphide oxidation (Polizzi et al. 2022a, 2022b). Alternatively, the reduced forms of sulphur present in the biogas could further be exploited for the removal of nitrate produced in a partial nitritation/anammox step (PN/A), through autotrophic denitrification (Grubba et al. 2022). Possible alternatives for innovative treatment lines are presented in figure S.2 (supplementary material).

The anammox process is considered a mature technology especially for highly N-loaded streams, but the effect of compounds present in industrial streams should be assessed prior to its application, since AMX activity could be dramatically affected (Carvajal-arroyo et al. 2013; Lackner et al. 2014). A successful implementation of PN/A process on Chromium-based tannery wastewater was presented by Frijters et al. (2007), reporting the pioneering treatment line implemented in the Lichtenvoorde (NL) wastewater treatment facility. Specifically, in the mentioned plant, an anaerobic IC® reactor allows for COD removal, sulfate reduction and biogas production; an aerobic sulphide oxidation reactor provides sulphide removal coupled with elemental sulphur recovery and last, a PN CIRCOX® reactor is coupled with an Anammox reactor for autotrophic N removal. An important milestone of their work was the achievement of stable operation in the anaerobic treatment unit, since the effectively treatment of the complex COD mixture present in tannery wastewater was not obvious.

In the newly conceived treatment alternatives, AMX process emerges a key process. To the best of our knowledge, there are no studies on the suitability of the anammox process for nitrogen removal in vegetable tannery wastewaters and the present study is intended to provide an evaluation of possible inhibitory effects on the anammox biomass. Recalcitrant tannin-related organic matters and salinity are selected as the two potential inhibitory factors and studied both for their separate and combined effect. Parallel batch tests with synthetic non-saline and saline solutions as well as real tannery wastewater were performed in order to assess the sole contribution of salinity and the aggregate effect of salinity and recalcitrant compounds.

## Material and methods

### *CuoiodepurWWTP*

In Cuoiodepur WWTP, more than 95% of the polluted load in terms of nitrogen, organic carbon, sulphur and salts is related to the industrial stream; whereas in terms of flowrate, the municipal stream accounts for around 40% of the total incoming flowrate. The almost 1:1 dilution is, in fact, a practical way to attenuate the potential inhibitory effect of high salinity and tannins levels, as well industrial load fluctuations. Currently, the actual salinity content of water entering the biological unit is 2–3 gCl/l and 1–1,5 gSO_4_^2−^/l, with an average electrical conductivity of 10 ± 2mS/cm (at 25 °C), whereas these parameters are almost double in the raw tannery influent, as presented in table 1 (see fig. S.1).

**Table 1:** Average characteristics of tannery wastewaters as reported in literature and observed in Cuoiodepur WWTP

Parameter (mg/l)	Reported TW^1^	Cuoiodepur TW^2^
COD	3000–23,000	12,800 ± 1370
TSS	2000–3000	5630 ± 1260
N–NH_4_^+^	120–250	320 ± 60
S^2−^	50–130	240 ± 100
Cl^−^	2000–7000	6030 ± 1120
SO4^−−^	1700–2700	12,800 ± 1370

^1^Mannucci et al. (2010); Zhao and Chen (2019)

^2^Industrial influent, average values over 2015–2019 (curtesy of Consorzio Cuoiodepur S.p.a)

### Batch activity tests

The objective of the experiment was to assess the effect of salinity and of (potentially) inhibitory organic compounds, such as tannins, separately.

The manometric batch test was implemented according to the general procedure reported by Lotti et al. (2012). Six OxiTop® bottles (each with a total volume of 350 ml, WTW, Germany) were run for a total of 8 days. Manometric tests allow for the estimation of anammox activity (i.e. nitrogen removal) through the monitoring of the headspace pressure increase due to N_2_ production. Three conditions were tested in duplicates: (i) fresh biomass suspended in synthetic medium, herein Control Test, CT; (ii) fresh biomass acclimated to saline conditions and suspended in synthetic saline medium, herein Saline Control Test, SCT; (iii) fresh biomass acclimated to saline conditions and suspended in pre-treated tannery wastewater, herein TWT. The experimental conditions are summarised in Table 2. Fresh AMX granular biomass was withdrawn from an AMX gas-lift reactor as reported in Polizzi et al. 2022a, 2022b, after 245 days of operation. AMX biomass used in SCT and TWT tests was preliminary acclimated to saline conditions, in order to avoid transient saline shock effect in the assessment of biomass activity. The procedure used for biomass acclimation is described in Sect “Biomass acclimation procedure to saline conditions”. Prior to the test, a mixture of N_2_/CO_2_ (95% and 5%, respectively) was sparged in the headspace for 5 to 10 min, in order to create anoxic conditions. Bottles were then placed in a pre-heated incubator at 30 °C. Continuous mixing was provided by an orbital shaker set at 150–180 rpm. Tests were run at 30 °C for three main reasons: (i) it is an optimum temperature for AMX activity; (ii) the biomass used in the test was grown at 30 °C; (iii) influent TW in Cuoiodepur WTTP typically exhibits warm temperatures and values of 25–35 °C are observed, on average, in the biological unit of the plant, throughout the year. Fresh biologically pre-treated tannery wastewater, herein tannery WW (TW), was withdrawn at the exit of the secondary effluent of Cuoiodepur WWTP and used in test TWT. Such a pre-treated wastewater was deemed the most suitable stream to study potential inhibitory effect on anammox biomass since the salt and tannin content is the same of the stream currently entering the biological unit and no readily biodegradable COD is present.

**Table 2:** Experimental conditions

Condition tested	Biomass	Liquid medium	Conductivity [mS cm^−1^ @25 °C]
Control Test, CT	Non-acclimated	Non-saline Synthetic Medium	4.3
Saline Control Test, SCT	Acclimated*	Saline Synthetic Medium	12
Tannery WW test, TWT	Acclimated*	Tannery WW	12

*To saline conditions according to the fast acclimation procedure (Sect “Biomass acclimation procedure to saline conditions”)

Tannery WW was filtered with paper filters in order to remove residual activated sludge in suspension. The amount of biomass placed in each bottle was set in order to achieve a complete nutrient removal within 7–12 h, since preliminary biomass activity tests results were available from Polizzi et al. (2022a, 2022b). Volatile Suspended Solids (VSS) analysis were done at the end of the test. The entire liquid content was filtered and incinerated. Average VSS concentration was 1.1 ± 0.3 g/l. Concentrated pulses of ammonium and nitrite were provided through addition of 1 M (NH_4_)_2_SO_4_ and 1 M NaNO_2_, in order to achieve 40–80 mgN/l both as ammonium and nitrite in the bulk liquid. A minimum of 1:1 ratio of ammonium:nitrite was ensured in order to obtain nitrite-limiting conditions. A total of 6 consecutive pulses were provided. Between spike 4 and 5, a mixture of N_2_ and CO_2_ (95% and 5%, respectively) was sparged in the headspace in order to avoid CO_2_ limitation drawbacks. Intermediate supernatant samples were taken between one spike and the subsequent in order to analyse ammonium, nitrite and nitrate concentration. pH was not controlled, but monitored throughout the tests. COD was also analysed in TWT. Analyses on the nitrogen components were used to perform nitrogen mass balances, comparing removed nitrogen (as N–NO_2_^−^) and N_2_ estimated by pressure increase. The stoichiometry from Lotti et al. (2014), presented in Eq. 1, was used to perform N balances for the anammox reaction.

1 $${NH}_{4}^{+}+1.225{NO}_{2}^{-}+0.073{HCO}_{3}^{-}+0.024{H}^{+}\to 1{N}_{2}+0.21 {NO}_{3}^{-}+0.073C{H}_{1.74}{O}_{0.31}{N}_{0.2}+1.95{H}_{2}O$$

Finally, in order to confirm the statistical significance of the data, paired t-test was performed on the paired SAA outcome sets (CT- SCT; CT-TWT; SCT-TWT). T-test on paired means was considered appropriate since it allows to assess whether the mean difference in the pairs is different from zero (McDonald 2014). The resulting p-value was calculated through the data analysis tool pack of Microsoft Excel (t-test, paired, double tale) and the significance level, α, set at 0.05.

#### Biomass acclimation procedure to saline conditions

The procedure described here was adopted in order to avoid saline shock effects on biomass activity. Tannery wastewater was withdrawn from the outflow of the biological unit (figure S.1) and characterized in terms of conductivity, chloride, sulphate, metals, COD, ammonium, nitrite and nitrate. The conductivity observed in tannery WW was as high as 10 mS/cm (at 25 °C) and mainly related to chlorine and sulphate ions. Synthetic mineral medium used to feed the source AMX gas-lift reactor held a background conductivity of 4.3 mS/cm (at 25 °C). A saline synthetic medium was prepared by adding chloride (as NaCl and KCl, in a 1:1 ratio) and sulphate (as Na_2_SO_4_) to the synthetic mineral medium in order to achieve the same electrical conductivity of tannery WW. The ratio Cl^−^/SO_4_^2−^ ions was kept as close as possible to the one observed in the real WW. A total of 10 saline solutions (around 300 ml each) were prepared by diluting the saline synthetic medium with different ratio of distilled water. Saline solutions had increasing conductivity levels starting from 5 mS/cm to 10 mS/cm (targeted conductivity, i.e. real wastewater conductivity), with a progressive increase of 0.5 mS/cm. All the solutions were kept at room temperature (21–24 °C). Fresh granular biomass was withdrawn from the anammox reactor and acclimated to room temperature in a 300 ml flask with (non-saline) synthetic medium. A step-wise exposure procedure towards increasing saline conditions was implemented. At each step, supernatant was removed and granular biomass poured into the saline solutions at increasing conductivity. The maximum conductivity increase at each step was around 0.5 mS/cm and biomass was left to acclimate for a minimum of 20 min prior to be poured in the saline solution with higher conductivity. Once the biomass was exposed for more than 20 min to the last saline solution (10 mS/cm) the acclimation procedure was considered concluded. The overall acclimation procedure lasted around 5.5 h.

The aliquot of the withdrawn biomass used to seed the non-saline control test was left at room temperature in non-saline synthetic medium during the duration of the acclimation procedure, in order to avoid activity discrepancies due to temperature gradients, between the acclimated and the not-acclimated biomass.

### Analytical*** methods***

Ammonium, nitrite, nitrate and COD were analysed spectrophotometrically through commercial kits (Dr. Hach Lange). Further analysis on Chloride, Sulphate and Phosphate ions were conducted through Ionic Chromatography, IC (Dionex ICS-1100, ThermoScientific). Metals in TW were analysed through inductively coupled plasma optical emission spectrometry, ICP-OES (Optima 2100 DV ICP-OES, PerkinElmer), after sample digestion with nitric acid (one-hour digestion at 100 °C, followed by filtration at 0.45 μm). TSS and VSS analysis were performed according to standard methods (APHA, 2005). Electrical conductivity was measured through a portable electrical conductivity meter (CM 35, Crison instrument), equipped with a temperature sensor (Pt 1000); the instrument provides conductivity values at 25 °C and all values reported in the present work are referred to this reference temperature.

## Results

### Batch activity tests

Table 3 reports the analytical composition of the pre-treated wastewater; pH was 7.5 and conductivity 10 mS/cm. The saline synthetic solution achieved a chloride and sulphate concentration of 2100 mgCl^−^/l and 1400 mgSO_4_^−−^/l, respectively. Chloride concentration was slightly lower than the one of the tannery WW, but sufficient to achieve the conductivity threshold of 10 mS/cm since the background conductivity of the synthetic medium was as high as 4 mS/cm. Analysis on metals concentration showed that none of the element presented inhibitory levels (Graaf et al. 1996; Huang et al. 2022). Note that the COD concentration of the pre-treated TW is related to recalcitrant colloidal organic compounds, mainly tannins, removed in the plant through tertiary chemical-physical treatments.

**Table 3:** Tannery (pre-treated) wastewater used in the TWT test

Parameter	Concentration [mg/l]
COD_tot_	451
COD_filtered_	400
N–NH_4_^+^	0.6
N–NO_2_^−^	< 0.1
N–NO_3_^−^	2.2
Cl^−^	2750
SO_4_^−^	1370
Cu	0.02
Cr	0.31
Ni	< DL*
Pb	0.5
B	0.5
Cd	< DL*
Al	0.11
Zn	0.17
Fe	1.51
P	0.47

*Below Detection Limit (DL)

**Fig. 1:**
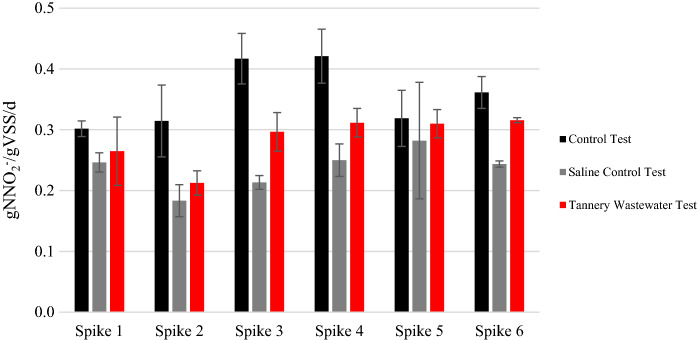
SAA results from six consecutive spikes of nutrients

Figure 1 shows the results of the maximum SAA values observed in the three tests, at each of the six consecutive spikes. Average values and standard deviation are reported for each duplicate.

The nitrogen balance closed within a 10% of error; pH remained at 7.3 ± 0.2 throughout the experiment, in all the tests. It can be observed that the control test (CT) keeps the highest activity throughout the experiment. SAA in CT showed its maximum values after spikes 3, 4 and 6, during which nitrite and ammonium concentrations were around 75 mgN/l, higher than in the other spikes where they reached around 40–50 mgN/l.

**Fig. 2:**
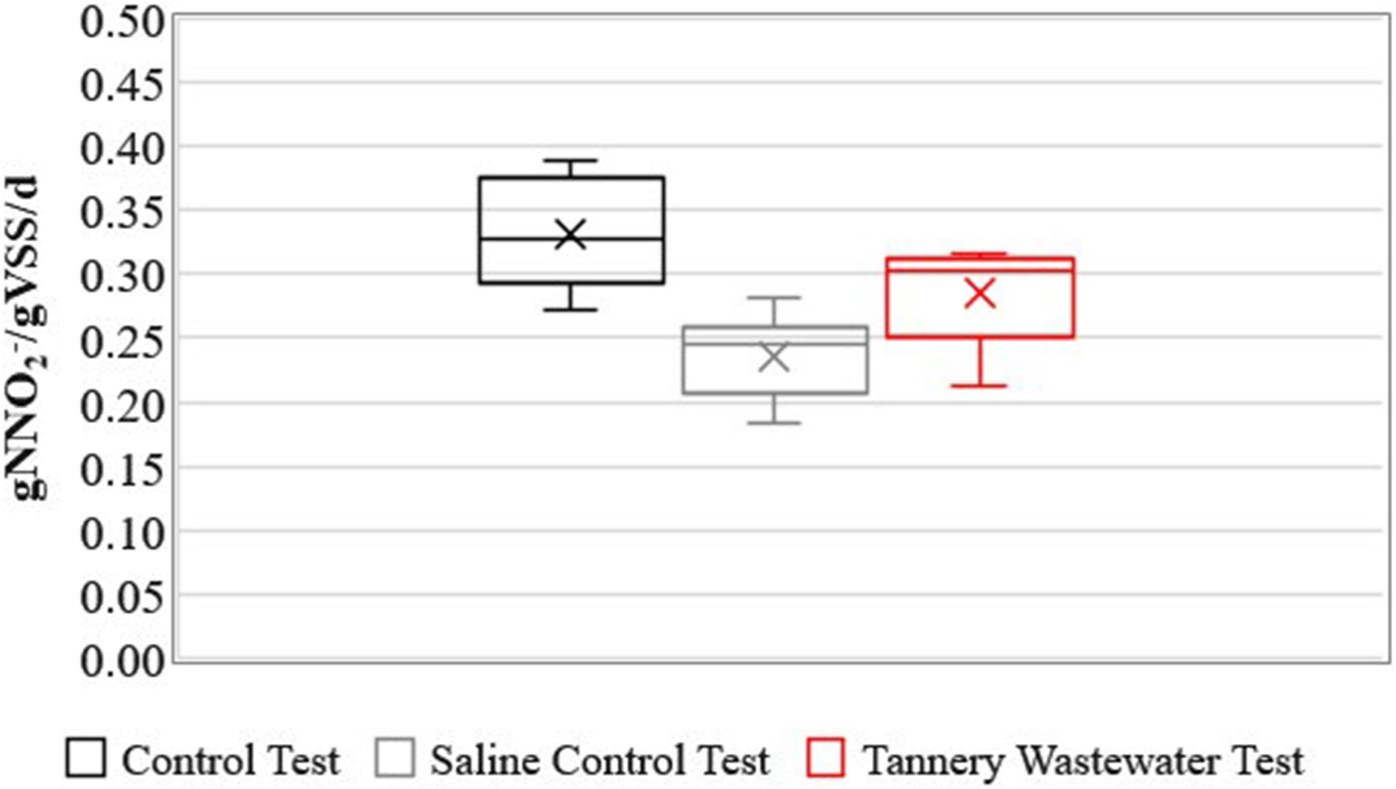
Box and Whisker plot of SAA activities for the three tested conditions (median, internal line; mean, internal cross)

In Fig. 2, Box and Whisker plot of the SAA values obtained in each of the three series of data is reported. Considering average values and comparing to non-saline control test, SAA in saline control test and tannery WW test showed a decrease of 28% and 14%, respectively. The stoichiometry observed in each test is reported in Table 4 (values estimated for spike 2, 4 and 6). The statistical analysis confirmed the statistical significance of the experimental outcomes, since the p-values fell in the range of 0.01–0.04, lower than the threshold α-value of 0.05 (0.0110, 0.0395 and 0.0069 for the pairs CT-SCT, CT-TWT and SCT-TWT, respectively).

**Table 4:** Observed stoichiometry in the inhibition experiment

Test	NO_3_^−^_prod_/NH_4_^+^_rem_	NO_2_^−^_rem_/NH_4_^+^_rem_
	mgN/mgN	mgN/mgN
CT	0.15 ± 0,06	1.44 ± 0.32
SCT	0.13 ± 0,02	1.42 ± 0.31
TWT	0.21 ± 0,13	1.61 ± 0.47

As it can be observed, TWT presents the highest variation either in NO_3_^−^/NH_4_^+^ ratio and in the NO_2_^−^/NH_4_^+^ one. Possibly, interference in the colorimetric analyses might have caused by the coloured matrix of TW. COD analyses, in the TWT duplicate, were performed prior to the 5th pulse and at the end of the test in order to assess possible heterotrophic denitrification. COD_filt_ resulted in 372 ± 4 and 364 ± 4 mg/l in the intermediate and final analysis, respectively. As the initial COD_filt_ was 400 mg/l, ca. 30 mg/l of COD_filt_ were removed at the initial and intermediate time, whereas COD remained quite stable from the 5th spike until the end of the test. Filtered COD of biologically pre-treated tannery WW is mainly related to dispersed or colloidal organic fractions, mainly tannins, a recalcitrant fraction that exits the biological unit, even under extensive aeration conditions (SRT in Cuoiodepur WWTP is as high as 70–80 d). In Cuoiodepur WWTP, such a high content in filtered COD is removed through chemical precipitation prior to effluent discharge. According with personal communication with plant process engineer, sudden COD depletion has been observed several times when pre-treated WW was dosed in activated sludge lab-scale reactors. Such a fast depletion has been ascribed to adsorption phenomena more than to fast biodegradation due to the recalcitrant nature of the suspended organic matter as well as the physical properties of colloidal particles. The evidence that COD_filt_ remains almost constant in the last two days of the test, is in line with such an assumption. Anyway, the evidence that average NO_2_^−^/NH_4_^+^ ratio in test TWT was higher than in test CT and SCT, suggests a possible impact of heterotrophic denitrification, leading to higher NO_2_^−^ removal than the stoichiometric one. Nevertheless, nitrate was not consumed but rather produced and NO_3_^−^/NH_4_^+^ ratio in TW was also higher than in the other tests. Denitrification in tannery wastewater has been reported to require COD/TKN ratio as high as 8–12 gCOD/gN, due to the presence of slowly biodegradable organic fractions (Carucci et al. 1999; Szpyrkowicz and Kaul 2004). Thereby, it can be speculated that 2–5 gCOD/gN-NO_2_^−^ would be required for denitritation only—considering the different electron requirement for nitrite reduction compared to complete nitrate reduction. According to such a ratio and the observed COD reduction, 5 to 8% of the nitrite dosed in the first fourth spikes could have been removed by heterotrophic denitritation. The corresponding rate of heterotrophic denitrification rate, cautiously calculated over the time of maximum N_2_ production rate, would have accounted for 6–9% of the observed rate, confirming that its possible impact in the calculation of the MSAA was marginal. The evidence that SAA observed in the last two spikes, when no significant COD removal was observed, was comparable to the one of the first spikes, further supports this hypothesis. On the other hand, the higher NO_2_^−^/NH_4_^+^ and NO_3_^−^/NH_4_^+^ ratios observed in TWT could have been also a result of additional NH_4_^+^ made available through ammonification of organic nitrogen present in the TW and not accounted in the ratios’ calculation. Likely, a partial contribution of the two phenomena of heterotrophic denitrification and organic nitrogen ammonification occurred.

Results from the inhibition experiments indicate that tannery wastewater does not show any severe inhibition effect on anammox granular biomass.

### Discussion

The results from the inhibition experiment suggest that the application of the anammox process to vegetable tannery wastewater has no technical limitation. According to the outcomes, salinity more than tannins seems to show a potential inhibitory effect.

The effect of salinity on anammox biomass has been extensively studied in the last decades, in order to assess its feasibility with saline N-rich wastewaters such as those generated by seafood, leather and textile dying industries, leachate as well as digestate from anaerobic digestion of organic fraction of municipal solid waste (OFMSW). Two main factors are reported to be crucial for stable treatment of saline wastewaters: (i) microbial population shift towards halophilic or more saline-tolerant species, such as “*Ca.* Scalindula” and “*Ca.* Kuenenia”; (ii) proper biomass acclimation to saline conditions. High salt concentrations may have detrimental effect on biological activity due to cascade mechanisms related to osmotic pressure on the cell wall, enzymatic inhibition and, ultimately, cellular lysis (Zhang et al. 2020). Stable anammox activity at saline conditions has been reported after biomass acclimation. A concentration of 30 gNaCl/l is pointed out by many authors as the salinity threshold for stable process performance (Dapena-Mora et al. 2010; Kartal et al. 2006; Li et al. 2018; Ma et al. 2012). In some cases, a conducive effect on SAA has been shown in case of gradual increase in salinity concentration until 20 gNaCl/l (Dapena-mora et al. 2010; Jin et al. 2011) or moderate salinity shock (Ma et al. 2012). On the contrary, in case of intense salinity shock events, a transient (and typically reversible) inhibition in anammox activity has been observed together with a progressive process recover, the recovery period being related to the saline gradient (Ma et al. 2012). Experiences with AMX granular biomass treating digestate of OFMSW showed 50% inhibition at 6 mS/cm (at 25 °C), i.e. IC_50_, for non-acclimated biomass (Scaglione et al. 2017) and 70% inhibition at 11.2 mS/cm (25 °C) for acclimated biomass (Lotti et al. 2019), yet allowing for stable performance in a continuous AMX reactor. As reported by the authors, these values are lower than the estimated threshold of 11.6 and 22.6 mS/cm (25 °C), corresponding to the IC_50_ salt concentrations of 5.4 gNaCl/l reported by Carvajal-arroyo et al. (2013) and 13.5 gNaCl/l reported by Dapena-mora et al. (2010). However, it should be noticed that IC_50_ values are affected by several factors, such as biomass adaptation and salinity exposure (gradual/shock). Tannery wastewater considered in the present study presented a chloride concentration of around 3 gCl^−^/l (equivalent NaCl concentration of ca 5 gNaCl/l), sulfate concentration of 1.47 g/l and conductivity of 10 mS/cm and the observed inhibition ranged from 14 to 28%. In the scenario of anammox process applied to treat only the industrial wastewater collected to Cuoiodepur WWTP (no dilution with municipal WW), chloride concentration would be around 6 gCl^−^/l, equivalent to ca 10 gNaCl/l. Dapena-Mora et al. (2010) and Kartal et al. (2006) reported a conducive effect of moderate salt concentrations (5–6 gNaCl/l) on not acclimated biomass, whereas Jin et al. (2011) reported the same positive effect on acclimated biomass and around 70% SAA reduction in not acclimated biomass after shock exposure of few days to the saline condition. In the present work, a moderate 28% SAA reduction was observed in the SCT, holding a comparable saline concentration as the mentioned studies. It owns to be mentioned that the biomass used in the present study was withdrawn from a reactor operating at a moderate conductivity of 4 mS/cm and this factor likely contributed to the low inhibition observed. Moreover, the acclimation procedure adopted in the present work appeared effective to prevent severe and transient SAA reduction due to saline shocks and that the observed reduction is likely to be further attenuated in case of long-term exposure to the moderate saline level of pre-treated tannery wastewater as reported by several works (Dapena-Mora et al. 2010; Ma et al. 2012). A longer adaptation period would have been conducive for a better biomass activity response in the inhibition experiment, since the acclimation time was much shorter than the one reported in similar studies (5.5 h versus 12 h to few days). Since the shock effect of sharp gradients in salinity concentration was not the objective of the present work, the fast acclimation procedure proved to be an effective solution to avoid transient responses to saline shocks. As presented in Polizzi et al. (2022a, 2022b), the inoculum presented a relative abundance of 25% for “*Ca*. Brocadia” and 8% for “*Ca*. Kuenenia”, resulting in a planctomycetes population composition of 68% for *Ca*. Brocadia and 13% for *Ca*. Kuenenia. The presence of the salinity-tolerant genus of “*Ca*. Kuenenia”, could have also favoured the positive response observed in the TWT.

Another interesting aspect of long-term salinity exposure is the impact on granules morphology and composition. Fang et al. (2018) and Zhang et al. (2020) recently published two comprehensive studies aimed at untangling mechanisms of anammox granulation response to high salinity levels. Both studies agreed in the general conclusion that high salinity levels (15 and 30 gNaCl/l) are not beneficial for granulation, due to many factors that can be synthetized as follows: (1) EPS composition is affected by salinity and, specifically, high salinity leads to higher production of the hydrophilic water-retaining polar molecules of polysaccharides (PS), and lower production of proteins (PN), showing an hydrophobic behaviour; (2) Ionic strength due to metal cations is usually reported as conducive to biomass aggregation into granules up to 0.1–0.2 M, though an opposite effect has been observed at higher values, possibly due to Na^+^ substitution to Ca^2+^ ion, the latter being involved in bridging mechanism within PS molecules.

The salinity levels considered by the two studies are much higher than the one considered in the tannery WW in Cuoiodepur WWTP, either pre-treated (and diluted) or not. Nevertheless, such evidences and possible mechanisms should be considered in case of real-scale implementation in order to ensure stable process performance.

## Conclusions

Results from batch tests on fresh biomass, acclimated to saline conditions, indicate that there is no evident inhibition of anammox biomass due to exposure to vegetable tannery wastewater. Salinity more than the mix of bio-refractory organic compounds is likely to be the impacting parameter for anammox biomass activity, since a 28% and 14% decrease in biomass activity was observed in the saline test and tannery WW test, compared to the non-saline control test. Nevertheless, biomass acclimation is widely reported to be effective to increase anammox activity under saline conditions and high anammox activities are likely to be achieved.

Further study on long-term exposure and salinity fluctuations, through continuous pilot-reactor operations are suggested as future developments. Analyses on microbial population as well as on granule’s morphology and EPS composition are recommended in order to produce comprehensive studies conducive for the real-scale implementation of the anammox process.

## Supplementary Information

Below is the link to the electronic supplementary material.

Supplementary file1 (PDF 213 KB)
