# Supplementary material for: Evaluating the suitability of granular anammox biomass for nitrogen removal from vegetable tannery wastewater
Source: Biodegradation. 2023 Feb 17;34(3):253–62. doi: 10.1007/s10532-023-10017-6 (PMC10148767; doi:10.1007/s10532-023-10017-6)
Supplement: Supplementary file 1 — Supplementary file1 (PDF 213 KB) [file 10532_2023_10017_MOESM1_ESM.pdf]

Evaluating the suitability of granular anammox biomass for nitrogen removal from vegetable tannery wastewater

Supplementary Material

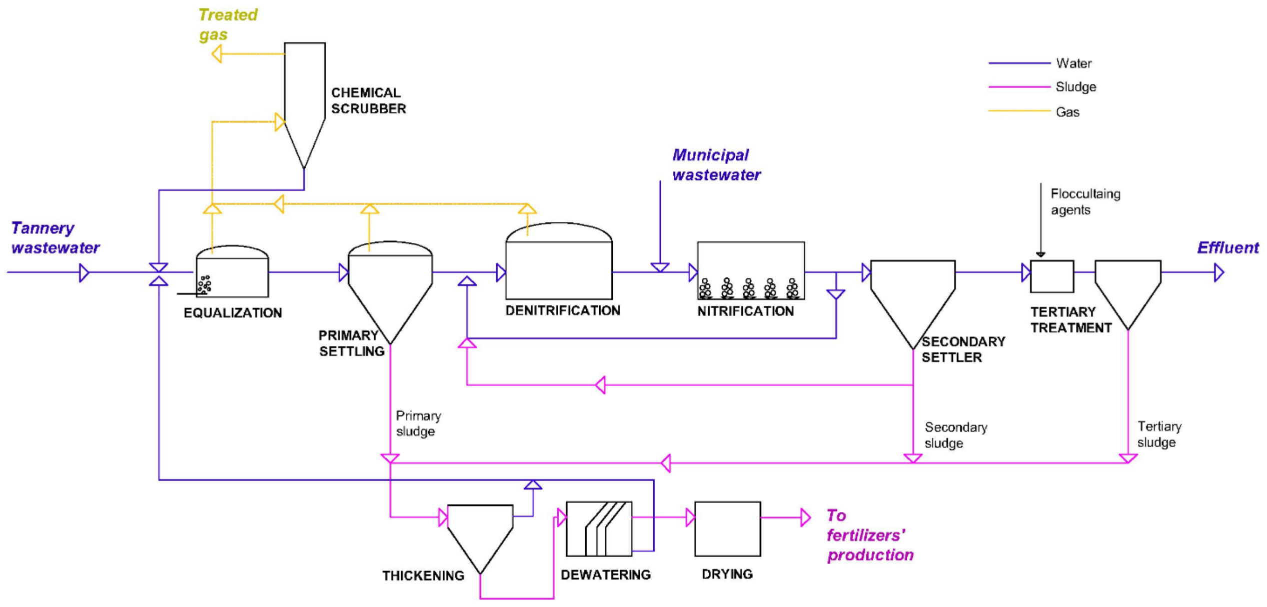

Figure S.1 Current configuration in the tannery wastewater treatment plant managed by Consorzio Cuoiodepur S.p.A. (San Miniato, PISA, IT).

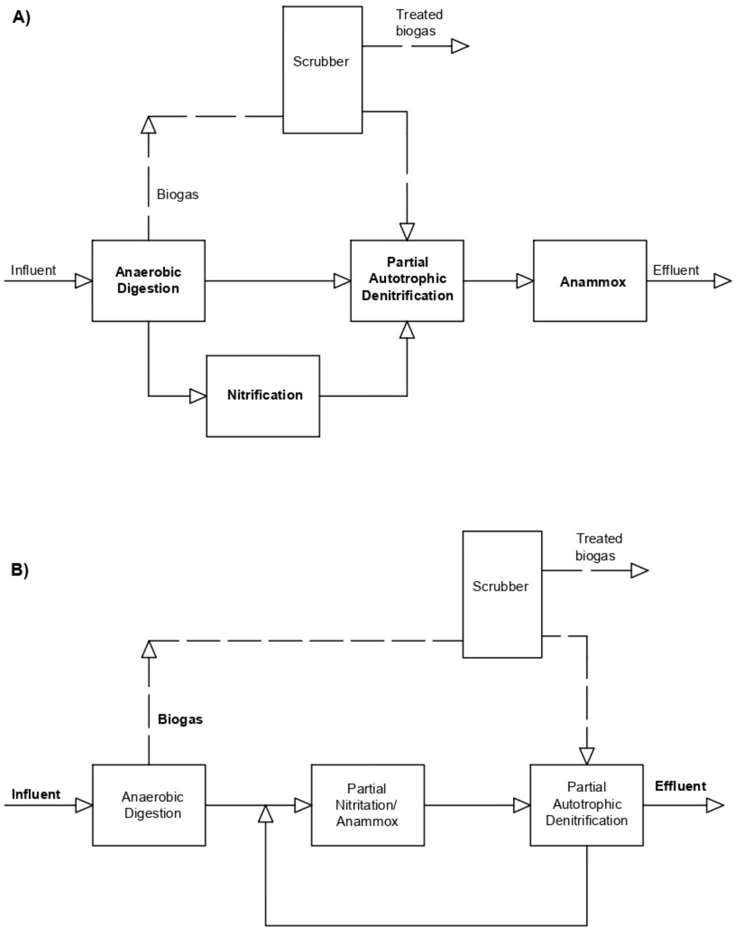

Figure S.2 Possible configurations for innovative treatment trains of tannery wastewater, integrating Carbon, Sulphur and Nitrogen cycles.
